# Advancing early identification of at-risk children: a consensus-based refinement and validation roadmap for the pediatric malnutrition screening algorithm

**DOI:** 10.3389/fnut.2026.1857903

**Published:** 2026-09-09

**Authors:** Robert D. Murray, Sanaa Y. Shaaban, Abdullah Shamsah, Ahmed Al Hammadi, Ahmed Amin, Ali Almehaidib, Ehab Khairy Elkhashab, Ekaterina A. Pyrieva, Entesar Al Hammadi, Funda Çetin, Khoula Al-Said, Muslim AlSaadi, Naglaa M. Kamal, Eslam Tawfik ElBaroudy

**Affiliations:** 1 Emeritus Professor of Pediatrics, Pediatric GI and Nutrition, The Ohio State University School of Medicine, Columbus, OH, United States; 2 Professor of Pediatrics, Department of Pediatrics, Consultant of Pediatric Clinical Nutrition, Ain Shams University, Cairo, Egypt; 3 President of Kuwait Pediatric Association, Kuwait, Kuwait; 4 Pediatric & Pediatric Emergency Consultant, Ministry of Health, Kuwait, Kuwait; 5 Dubai Health Authority, Dubai Medical College, Dubai, United Arab Emirates; 6 Abbott Laboratories, Dubai, United Arab Emirates; 7 King Faisal Specialist Hospital and Research Centre, Riyadh, Saudi Arabia; 8 Pediatric Clinical Nutrition Unit, Ain Shams University, Cairo, Egypt; 9 Federal Research Centre of Nutrition, Biotechnology and Food Safety, Moscow, Russia; 10 Ege University Faculty of Medicine, Department of Pediatrics, İzmir, Türkiye; 11 Department of Paediatric Gastroenterology, Royal Hospital, Muscat, Oman; 12 Department of Pediatrics, College of Medicine, King Saud University, Riyadh, Saudi Arabia; 13 Department of Pediatrics and Pediatric Hepatology, Kasr Al-Ainy Faculty of Medicine, Cairo, Egypt; 14 Department of Pediatrics, Faculty of Medicine, University of Suez, Suez, Egypt; 15 Department of Health, Kids Heart Medical Center affiliated Columbia University, Abu Dhabi, United Arab Emirates

**Keywords:** content validity, early detection, growth monitoring, malnutrition, Middle East, pediatric nutrition, screening, Z-score

## Abstract

**Introduction:**

Mild malnutrition risk remains an underrecognized yet clinically important contributor to impaired growth and development in children aged 1–10 years across the Middle East. Regional estimates indicate that 15–25% of children in the Gulf Cooperation Council (GCC) fall within the mild malnutrition risk category (Weight-for-Height Z-score [WHZ] between −1 and −2 SD), a subgroup vulnerable to progressive growth faltering yet excluded from conventional malnutrition diagnostic thresholds (WHZ < −2 SD). The Pediatric Malnutrition Screening Algorithm (PMSA-2025) was refined through a structured regional expert consensus process to enable early, standardized detection of these at-risk children in outpatient and community healthcare settings.

**Methods:**

A 14-member multidisciplinary expert panel from different countries (KSA, UAE, Egypt, Kuwait, Oman, Russia, United States, and Türkiye) refined the PMSA-2025 using a three-round, hybrid modified Delphi approach conducted in September 2025. Expert selection criteria included a minimum of 10 years of clinical or academic experience in pediatric nutrition or gastroenterology, active involvement in malnutrition-related clinical practice or research, and representation of diverse regional healthcare contexts. Content validity was assessed using the Item-level Content Validity Index (I-CVI), with a threshold of ≥0.80 for domain retention. Six clinical domains were retained from an initial pool of eight candidate domains after Delphi-based consensus: perinatal history, growth trajectory, prior nutritional diagnosis, hospitalization frequency, infection recurrence, and feeding behavior. Each domain was operationalized with measurable, age-appropriate criteria and incorporated into a binary (Yes/No) scoring system (total score: 0–6). The tool is designed for use by pediatricians, family physicians, nurses, and trained community health workers in children aged 1–10 years with WHZ between −1 and −2 SD. Exclusion criteria include children with primary chronic systemic disease driving growth impairment and those already receiving formal nutritional support.

**Results:**

All six retained domains achieved an Item-level CVI (I-CVI) ≥ 0.85 in the final consensus round. Two initial candidate domains (dietary diversity score, caregiver nutritional literacy) were excluded due to insufficient agreement (I-CVI < 0.70). Three domains required definitional refinement during Round 2, achieving ≥90% final agreement in Round 3. The PMSA-2025 stratifies mild malnutrition risk across three tiers: low (0–1), moderate (2–3), and high (4–6). It requires no specialized equipment beyond a standard scale and can be completed within routine consultations.

**Conclusion:**

The PMSA-2025 represents a consensus-based, multidimensional screening tool with established content validity designed to address the unmet need for early mild malnutrition risk identification in outpatient and community pediatric settings across the Middle East.

## 1 Introduction

Childhood malnutrition remains a major global public health concern (1). While coordinated initiatives have significantly reduced severe malnutrition caused by starvation or chronic disease, mild-to-moderate undernutrition persists as an overlooked but clinically important problem (2). Nutritional deficits during infancy and early childhood impair growth, cognitive development, immune function, and overall human capital formation (3).

According to the World Health Organization (WHO), moderate malnutrition is defined as a Weight-for-Height Z-score (WHZ) of less than −2 standard deviation (SD) (4). Increasing evidence highlights that children within the mild-risk category (−1 to −2 SD) represent a critical target for preventive intervention (5). These children often exhibit early signs of growth faltering, inadequate dietary intake, or recurrent infections, yet they remain outside the formal diagnostic threshold for moderate-to-severe malnutrition (6). Without timely recognition and intervention, many children may progress to stunting, wasting, or long-term developmental impairment (7).

In the Middle East specifically, the burden of malnutrition encompasses both undernutrition and micronutrient deficiency despite apparent normal body weight. According to UNICEF State of the World’s Children data (2023), stunting prevalence among under-5 children ranges from approximately 8% in GCC countries to 15% in Egypt. Wasting affects 5–12% of under-5 children across the region. Critically, children in the mild risk range (WHZ − 1 to −2 SD) are estimated to constitute an additional 15–25% of the under-5 population across GCC and North African countries, a group not captured by conventional diagnostic thresholds but at meaningful risk of nutritional decline (8, 9). This disproportionate burden of ‘hidden’ malnutrition is compounded by persistent micronutrient deficiencies in iron, zinc, iodine, and vitamin A even among children with apparently normal body weight (8).

Rapid urbanization, adoption of Western dietary habits, and limited caregiver awareness of balanced nutrition have contributed to diets high in calories but low in essential nutrients (10). This pattern, coupled with increasing sedentary behavior and shorter breastfeeding duration, has led to the coexistence of undernutrition and obesity within the same populations (11).

Despite standardized WHO growth charts, identification of mild malnutrition risk in clinical settings remains inconsistent. Studies show that fewer than half of pediatricians routinely calculate or interpret Z-scores (12). In many outpatient and community clinics, fragmented follow-up and time constraints obstruct early detection of mild malnutrition risk (13). These gaps highlight the need for a simple, standardized screening approach that complements anthropometric evaluation and can be readily implemented across diverse healthcare environments.

To address these challenges, the Kingdom of Saudi Arabia Advisory Board on Pediatric Nutrition (KSA-ABPN) initiated the development of the Pediatric Malnutrition Algorithm in 2024, providing an initial framework for systematic risk assessment (9). However, the absence of a structured scoring system and variability in clinical interpretation limited its practical use. In 2025, a regional initiative was undertaken to refine the algorithm for broader clinical applicability. The present expert consensus document summarizes this refinement process and presents the resulting PMSA-2025 framework and prospective validation study roadmap.

## 2 Methodology

### 2.1 Target population and evidence review

The PMSA-2025 is designed for children aged 1–10 years, consistent with the WHO malnutrition surveillance framework. The tool is intended for deployment in primary care outpatient clinics, community health centers, and secondary-level pediatric outpatient departments across the Middle East.

Weight-for-Height Z-score (WHZ) was selected as the primary anthropometric anchor for this tool because it is the WHO-recommended indicator for detecting acute undernutrition and is the standard metric embedded in regional clinical practice and surveillance programs across the Middle East. While BMI-for-age is increasingly used for children above 5 years of age in some settings, WHZ remains appropriate and operationally consistent within the mild-risk screening context of this tool (1--10 years), particularly in outpatient and community settings where WHZ is routinely applied in growth chart practice. The mild-risk threshold of WHZ between −1 and −2 SD was selected because this range captures children who fall below the population norm but do not yet meet the diagnostic criterion for moderate malnutrition (WHZ < −2 SD), representing a clinically important but underserved prevention window. For children aged 6 years and above, where BMI-for-age may be considered complementary, the six PMSA-2025 clinical domain questions remain valid as behavioral and clinical risk indicators independent of the specific anthropometric index used. The appropriateness of WHZ versus alternative indicators across the full age range will be prospectively evaluated in the planned validation study.

Inclusion criteria: Children aged 1–10 years presenting for routine or illness-related consultations and WHZ between −1 and −2 SD (mild malnutrition risk range per WHO classification).

Exclusion criteria: Children with confirmed chronic systemic disease known to primarily drive growth impairment (cystic fibrosis, inflammatory bowel disease, congenital heart disease, chronic kidney disease); children already enrolled in formal nutritional support or therapeutic feeding programs; children with WHZ below −2 SD (those who should be managed under established moderate-to-severe malnutrition protocols).

The refinement process began with a structured targeted literature review of clinical, behavioral, and biological indicators predictive of mild malnutrition risk in early childhood. Searches were conducted across PubMed and Embase using terms related to pediatric malnutrition screening, growth faltering, mild undernutrition, and pediatric nutrition in the Middle East, covering publications from 2000 to 2024. Insights from this review guided domain selection and threshold definitions, with emphasis on evidence applicable to regional growth patterns, disease prevalence, and dietary behaviors.

### 2.2 Expert panel selection and refinement process

The consensus panel comprised 14 pediatricians and clinical nutrition specialists from different countries: KSA, UAE, Egypt, Kuwait, Oman, Russia, United States, and Türkiye. Expert selection followed a systematic nomination process based on the following criteria: (1) a minimum of 10 years of clinical or academic experience in pediatric nutrition or gastroenterology; (2) active involvement in malnutrition-related clinical practice or research within their country; (3) recognized standing within national or regional pediatric nutrition societies or policy bodies; and (4) representation of diverse geographic, clinical, and healthcare-system contexts to ensure regional generalizability.

The refinement followed a structured, three-round hybrid modified Delphi approach conducted in September 2025. Content validity was assessed using the Item-level Content Validity Index (I-CVI), calculated as the proportion of experts rating each domain as relevant or highly relevant (score ≥3 on a 4-point relevance scale). Domains achieving, I-CVI ≥ 0.80 were retained without modification; those with I-CVI 0.70–0.79 were revised; and those with I-CVI < 0.70 were excluded or merged.

Round 1: All 14 experts independently reviewed eight candidate domains from the 2024 model via structured electronic surveys. Responses were collected anonymously to minimize social desirability bias. Each domain was evaluated for clinical clarity, sensitivity, and feasibility in busy outpatient environments.

Round 2: Findings were summarized and presented at a moderated in-person consensus meeting. Domains with I-CVI < 0.80 were discussed and revised collaboratively. Three domains required refinement: growth trajectory (thresholds stratified by age), infection frequency (thresholds stratified by age), and hospitalization criteria (dual threshold added).

Round 3: The revised draft was electronically recirculated for final approval, with consensus defined as ≥80% agreement (≥ 11/14 experts). All six retained domains achieved final I-CVI ≥ 0.85 and ≥90% agreement. Anonymous responses were collected to ensure independent judgment.

While the PMSA-2025 development did not formally adopt the full COSMIN (COnsensus-based Standards for the selection of health Measurement Instruments) or CREDES (Consensus REporting of DEvelopment of Screening tools) frameworks (both optimally applied during prospective validation), key principles from both guided this refinement phase: expert panel composition and diversity, content validity assessment via I-CVI, structured iterative review, and transparent consensus thresholds. Regarding the role of the industry sponsor Abbott): funding was provided for the meeting site and participant honoraria. The sponsor did not participate in the design of Delphi survey instruments, the conduct of consensus rounds, the adjudication of disagreements, the drafting of the manuscript, or any editorial decisions. All content, domain selection, scoring rationale, and conclusions were determined exclusively by the expert panel and authors, and the final manuscript was reviewed and approved by all authors independently of the sponsor.

### 2.3 Scoring system and interpretation

A binary scoring system was established to maximize simplicity and reproducibility. The binary format was selected over Likert-type or weighted scoring based on three evidence-based rationales: (1) binary formats demonstrate superior inter-rater reliability in outpatient pediatric screening; (2) they minimize cognitive burden in high-volume settings; and (3) they allow straightforward integration into electronic health records and mobile applications. Each affirmative response was assigned one point (Yes = 1, No = 0), with a cumulative score of 0–6, where higher totals indicate greater nutritional vulnerability. The scoring complements anthropometric assessment rather than replacing it.

### 2.4 Expert validation and ethical considerations

The refined tool underwent multidisciplinary face validity review by pediatricians and nutrition specialists. Revisions focused on aligning terminology with local clinical practice, ensuring cultural appropriateness, and optimizing question flow for efficient use during pediatric consultations. The final six-item format was intentionally designed to allow completion by trained non-physician personnel (nurses, community health workers) without specialized resources. This refinement process did not involve direct patient participation or clinical data collection; ethical approval was therefore not required.

## 3 Results

### 3.1 Overview of consensus outcomes

The three-round Delphi process resulted in the PMSA-2025: a unified six-domain screening tool. The initial candidate pool contained eight domains (Table 1). Following the consensus process, two domains were excluded due to insufficient agreement: dietary diversity score (I-CVI = 0.57), which was excluded as it requires food frequency assessment considered resource-intensive for routine outpatient use and lacks a binary operationalization; and caregiver nutritional literacy (I-CVI = 0.64), excluded due to the absence of a standardized, region-specific definition agreeable to binary scoring.

**Table 1:** Consensus outcomes by domain: PMSA-2025 Delphi process.

Domain	Round 1 I-CVI	Outcome	Round 3 I-CVI	Final decision
Premature birth/low birth weight	0.93	Retained, minor wording clarified	0.93	Included
Has this child shown poor weight gain or growth faltering in the past 2 months (if <2 yrs) or 6 months (if ≥2 yrs)?	0.79	Revised: thresholds stratified by age (<2 yrs./≥2 yrs)	0.93	Included
Previous diagnosis/treatment of undernutrition	0.86	Retained, exclusion of chronic disease clarified	0.86	Included
Has this child been hospitalized more than twice (each >2 days) or once for >3–5 days in the past 6 months?	0.75	Revised: dual threshold added (>2 admissions OR single >3–5 days)	0.90	Included
Has this child had recurrent infections (>3 episodes in 2 months if <2 yrs.; or >3 episodes in 6 months if ≥2 yrs)?	0.79	Revised: thresholds stratified by age (<2 yrs. / ≥2 yrs)	0.90	Included
Has this child shown selective or altered eating behavior (reduced intake or food variety for ≥7 consecutive days, or consuming <75% of typical meal volume for at least 1 week)?	0.86	Retained, 7-day duration criterion added	0.86	Included
Dietary diversity score	0.57	Excluded: resource-intensive, lacks binary operationalization	—	Excluded
Caregiver nutritional literacy	0.64	Excluded: no standardized definition available	—	Excluded

I-CVI, Item-level content validity index. Threshold for retention: ≥0.80. Domains achieving, I-CVI 0.70–0.79 were revised; <0.70 were excluded.

Six domains were retained, all achieving final I-CVI ≥ 0.80. Three required definitional revision during Round 2 (growth trajectory, infection frequency, hospitalization criteria) and achieved ≥90% final agreement in Round 3. The binary scoring format (Yes = 1; No = 0) and the three-tier risk classification (Low: 0–1; Moderate: 2–3; High: 4–6) were approved with 100% (14/14) consensus.

### 3.2 Finalized screening domains

**Figure 1:**
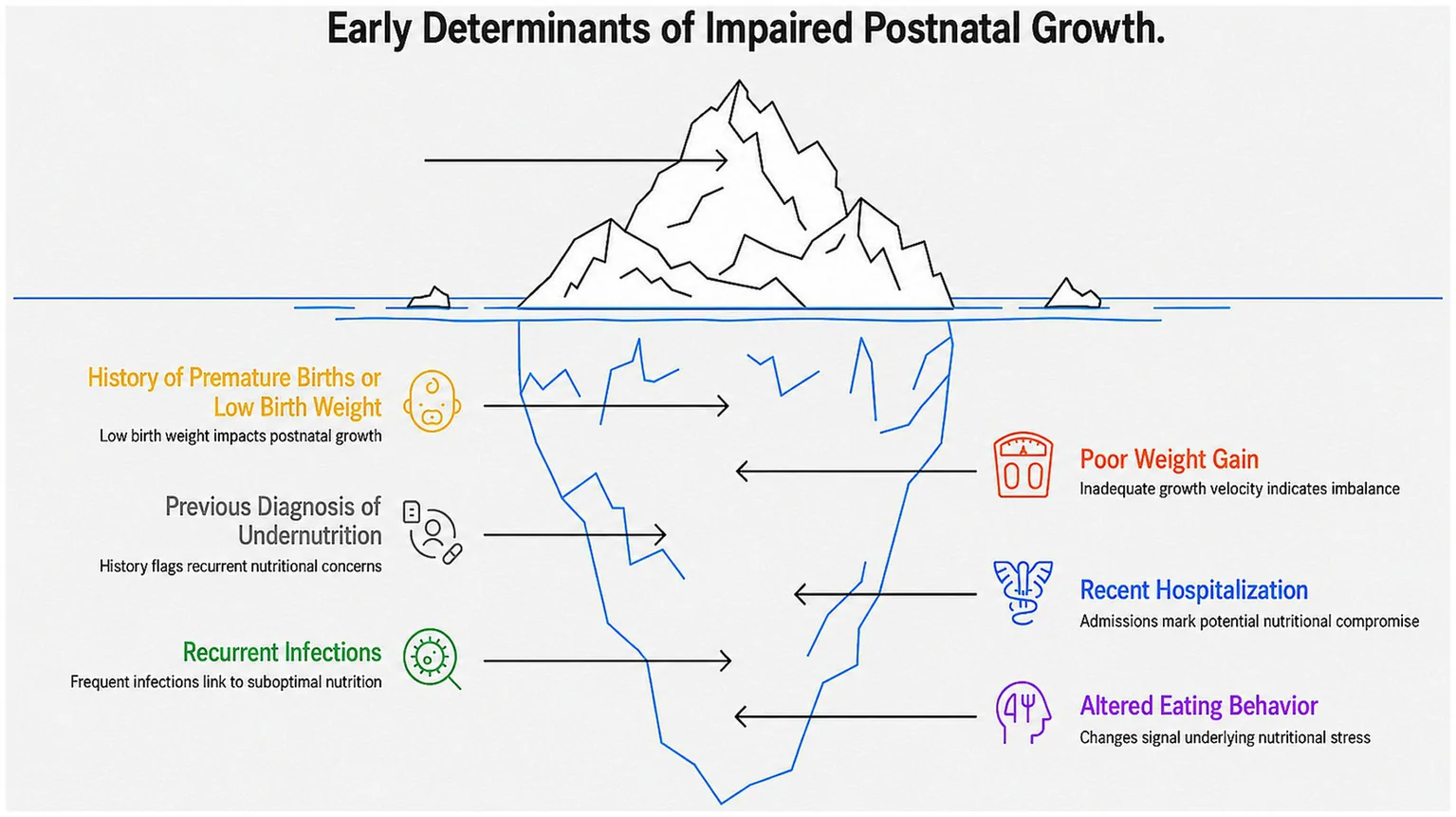
Early determinants of impaired postnatal growth.

The PMSA-2025 comprises six clinical questions designed for rapid completion during routine outpatient consultations (Figure 1; Table 2).

**Table 2:** Pediatric Malnutrition Screening Algorithm (PMSA-2025): Structure and scoring system.

Domain	Screening question	Definition/Clinical criterion	Scoring (Yes = 1; No = 0)	Clinical rationale/Reference
1	Was this child born prematurely (<37 weeks) or with low birth weight (<2.5 kg)?	Birth weight < 2.5 kg or < 37 weeks’ gestation		Low birth weight and prematurity are established predictors of early growth faltering and later undernutrition (26).
2	Has this child shown poor weight gain or growth faltering in the past 2 months (if <2 yrs) or 6 months (if ≥2 yrs)?	Growth velocity below expected range over 2 months (<2 yrs) or 6 months (≥2 yrs)		Slow growth velocity signals early malnutrition risk and predicts future stunting or wasting (26).
3	Has this child previously been diagnosed with or treated for undernutrition?	Documented nutritional deficiency or prior intervention		A prior malnutrition diagnosis indicates persistent or recurrent malnutrition risk and justifies continued monitoring (27).
4	Has this child been hospitalized more than twice (each >2 days) or once for >3–5 days in the past 6 months?	>2 hospitalizations for > 2 days each within past 6 months		Hospitalization reflects acute illness, catabolic stress, and reduced intake, all linked to deteriorating nutrition (28).
5	Has this child had recurrent infections (>3 episodes in 2 months if <2 yrs.; or >3 episodes in 6 months if ≥2 yrs)?	>3 episodes in 2 months (<2 yrs) or > 3 episodes in 6 months (≥2 yrs)		Frequent infections exacerbate nutrient loss and impair appetite, perpetuating the infection malnutrition cycle (29).
6	Has this child shown selective or altered eating behavior (reduced intake or food variety for ≥7 consecutive days, or consuming <75% of typical meal volume for at least one week)?	Recent reduction in intake, appetite, or food variety		Reduced intake or food selectivity contributes directly to inadequate energy and micronutrient supply (30).

Total score = sum of “Yes” responses (0–6).

Preliminary risk classification: 0–1 = Low risk; 2–3 = Moderate risk; 4–6 = High risk.

#### 3.2.1 History of premature birth or low birth weight

Low birth weight (<2.5 kg) or prematurity (<37 weeks gestation) was retained as the first domain. This criterion identifies children with early biological risk for impaired growth and subsequent undernutrition.

#### 3.2.2 Poor weight gain

For children under 2 years, poor weight gain was defined as a weight velocity below the 25th percentile for a defined age and sex over a two-month period, or a decline of ≥0.5 Z-score on the WHO weight-for-age chart. For children aged 2 years and above, the observation period was extended to 6 months.

#### 3.2.3 Previous diagnosis or treatment of undernutrition

A documented history of nutritional deficiency or prior intervention was included to flag children with recurrent or unresolved nutritional concerns. Chronic conditions known to independently drive growth impairment were explicitly excluded to ensure the tool focuses on nutritional rather than disease-driven causes.

#### 3.2.4 Recent hospitalization

The threshold was defined as either (a) more than two hospitalizations, each lasting over 2 days, within the preceding 6 months, or (b) a single hospitalization exceeding 3–5 days within the preceding 6 months. Short emergency visits are excluded.

#### 3.2.5 Recurrent infections

The threshold was set at more than three episodes within 2 months for children under 2 years, and more than three episodes within 6 months for those aged 2 years and above.

#### 3.2.6 Selective or altered eating behavior

Altered eating behavior was defined as a reduction in usual intake or food variety sustained for ≥7 consecutive days, or caregiver-reported consumption of <75% of typical meal volume for at least 1 week, distinguishing persistent intake reduction from transient picky eating.

### 3.3 Clinical application and interpretation

In clinical use, the PMSA-2025 functions as an adjunct to WHZ evaluation, stratifying children within the WHO-defined mild-risk range (−1 to −2 SD) into subcategories of higher or lower vulnerability (Table 2). A score of 0–1 indicates minimal concern and routine follow-up; 2–3 suggests moderate risk requiring closer monitoring or dietary counseling; and 4–6 identifies children at high risk who may benefit from proactive nutritional support including oral nutrition supplementation (ONS). All cut-off values are provisional and will be empirically confirmed through the planned prospective validation study.

## 4 Discussion

The present consensus describes a practical screening tool and a pre-specified validation roadmap for the PMSA-2025, designed to standardize early identification of mild malnutrition risk in children aged 1–10 years across Middle Eastern outpatient and community settings.

### 4.1 Significance of early identification

Mild undernutrition (WHZ − 1 to −2 SD) has traditionally received limited clinical attention despite growing evidence linking early growth faltering with adverse developmental outcomes (14, 15). Children in this zone are at heightened vulnerability for future wasting, stunting, recurrent illness, and micronutrient deficiencies if unrecognized (14, 16). The PMSA-2025 advances preventive care by establishing operational thresholds for early risk anticipation, aligning with WHO and UNICEF priorities advocating early screening as the cornerstone of child growth promotion (17, 18).

### 4.2 Integration of biological and behavioral indicators

Traditional screening tools focus primarily on anthropometric measurements, which can miss early subclinical risk factors (19, 20). The inclusion of perinatal history, feeding behavior, and infection frequency in the PMSA-2025 enables a more holistic assessment of nutritional vulnerability (21, 22). Low birth weight and prematurity identify children with inherent metabolic disadvantages; repeated infections and hospitalizations serve as markers of physiological stress and potential nutrient depletion (23); and altered eating behavior reflects evolving insufficiency or psychosocial barriers to adequate intake (24).

### 4.3 Rationale for the six-domain approach

The six-domain structure reflects the complex, multifactorial etiology of childhood undernutrition. Integrating biological, clinical, and behavioral parameters enables recognition of subtle but cumulative risk patterns that no single parameter captures independently. The binary scoring format minimizes inter-rater variability and allows efficient completion during high-volume outpatient consultations.

### 4.4 Comparison with existing tools and advantages over conventional cut-offs

Existing pediatric malnutrition screening tools: STRONGkids, PYMS, and STAMP, have demonstrated reliability in inpatient hospital settings (25). It should be noted, however, that these tools were developed for different clinical contexts and age groups, and direct comparisons should be interpreted cautiously. None of the three tools was specifically designed for outpatient or community use targeting the mild-risk range (WHZ − 1 to −2 SD), nor do they incorporate behavioral or perinatal screening items; however, whether the inclusion of such domains in the PMSA-2025 translates into superior clinical performance remains to be established through prospective validation. Table 3 provides a structured contextual comparison.

**Table 3:** Comparison of PMSA-2025 with established pediatric malnutrition screening tools.

Feature	PMSA-2025	STRONGkids	PYMS	STAMP	Notes
Primary setting	Outpatient/community	Inpatient	Inpatient	Inpatient	PMSA-2025 designed for ambulatory care; inpatient use not evaluated
Target age	1–10 yrs	1–18 yrs	1–16 yrs	2–17 yrs	PMSA-2025 focused on highest-risk developmental window
Targets mild risk (WHZ − 1 to −2)	Yes	No	No	No	Not a feature of existing tools; prospective confirmation needed
Requires anthropometric equipment	Scale + stadiometer/length board	Scale + length board	Scale + height	Scale + height	PMSA-2025 minimal equipment requirement
Includes behavioral indicators	Yes	No	No	No	Behavioral domain absent from compared tools; sensitivity to be validated
Includes perinatal history	Yes	No	No	No	Perinatal domain absent from compared tools; clinical utility to be confirmed
Binary scoring	Yes	Partial	No	Partial	Binary format may reduce inter-rater variability; to be confirmed in validation
Prospectively validated	Planned	Yes	Yes	Yes	Prospective validation is planned; not yet initiated
COSMIN principles applied	Partial (content validity phase)	Yes	Yes	Yes	Full COSMIN adherence planned; partial adherence in current phase only

PYMS, Paediatric Yorkhill malnutrition score; STAMP, Screening tool for the assessment of malnutrition in paediatrics; STRONGkids, Screening tool risk on nutritional status and growth; COSMIN, COnsensus-based Standards for the selection of health measurement instruments.

The conventional WHZ − 2 SD diagnostic threshold identifies established moderate malnutrition but systematically excludes children in the −1 to −2 SD range who are at meaningful risk of progression. The PMSA-2025 adds a multidimensional clinical and behavioral overlay to this anthropometric window, enabling risk stratification within this ‘gray zone’ that conventional thresholds fail to address. For example, a child with WHZ − 1.5 SD, a history of prematurity, recurrent infections, and recent reduced oral intake scores 3/6 (moderate risk) and warrants proactive monitoring, whereas the same WHZ in isolation would prompt no formal intervention under current guidelines.

### 4.5 Contribution to pediatric practice

The PMSA-2025 bridges an important operational gap between nutritional screening and clinical decision-making. Its design empowers general pediatricians, family physicians, nurses, and trained community health workers to detect malnutrition risk proactively, even in settings where formal nutrition assessment tools are unavailable. Its concise format supports integration into electronic medical records and mobile applications for longitudinal monitoring.

### 4.6 Strengths, limitations, and future directions

The PMSA-2025 has several notable strengths related to its development and design. First, it was developed by a multidisciplinary expert panel of 14 specialists from different countries, providing broad regional representation across diverse clinical and healthcare-system contexts. Second, the structured three-round hybrid Delphi methodology with I-CVI-based quantitative agreement assessment (I-CVI threshold ≥0.80) provides a transparent and reproducible foundation for content validity. Third, the binary scoring format is optimized for high-volume outpatient settings and requires no specialized equipment beyond standard anthropometric tools (scale and stadiometer/length board), lowering the barrier to routine implementation. Fourth, to our knowledge, the PMSA-2025 is the first screening tool specifically designed to target the mild malnutrition risk category (WHZ − 1 to −2 SD) for outpatient and community use in a Middle Eastern context. Finally, the integration of behavioral, perinatal, and clinical indicators enables the detection of early nutritional risk that is not captured by anthropometry alone or by existing inpatient-focused tools such as STRONGkids, PYMS, and STAMP.

## 5 Limitations

The PMSA-2025 represents a consensus-based framework in the content validity phase of development. It is important to emphasize that no patient-level data have been collected to date. Accordingly, no diagnostic accuracy parameters (sensitivity, specificity, PPV, NPV, ROC/AUC), predictive validity estimates, or reliability coefficients (inter-rater, test–retest) are currently available for this tool. All risk stratification thresholds are based exclusively on expert consensus and must be regarded as provisional until confirmed through the planned prospective validation study. Additional limitations include: (1) The evidence review was a structured targeted review rather than a formal systematic review or scoping review pre-registered with PROSPERO; formal PRISMA-ScR or Cochrane methodology was not applied, which limits reproducibility of the evidence synthesis. (2) All three risk classification tiers (Low: 0–1; Moderate: 2–3; High: 4–6) and their corresponding scoring thresholds are provisional and subject to empirical refinement through the planned prospective validation study. (3) While COSMIN and CREDES principles informed key aspects of development, full adherence to these frameworks was not achievable in this consensus phase. (4) Expert panels funded by industry carry a risk of commercial bias, which we acknowledge; all content was reviewed and approved by all authors independently of the sponsor.

## 6 Future directions

A prospective multicenter validation study is planned across specific centers in different countries (KSA, UAE, Egypt, Kuwait, Oman, and Russia) with a target enrollment of approximately 1,200–1,300 children aged 1–10 years with WHZ between −1 and −2 SD in primary care and community settings. The study will assess sensitivity, specificity, PPV, NPV, ROC/AUC curves, inter-rater reliability (Cohen’s kappa), test–retest reliability, and predictive validity at 3 and 6-month follow-up. Validated Arabic and Turkish translations of the PMSA-2025 tool will also be developed to assist in the study. The resulting data will inform refinement of scoring thresholds and guide integration into regional pediatric electronic health platforms.

### 6.1 Broader public health implications

Early detection and targeted nutritional intervention are central to achieving the United Nations Sustainable Development Goal 2 and WHO’s 2025 Global Nutrition Targets to reduce stunting and wasting, alongside the region’s growing burden of childhood obesity and persistent micronutrient deficiencies such as iron, iodine, and vitamin A (31, 32). By operationalizing mild-risk identification, the PMSA-2025 contributes to a prevention-oriented model that can prevent progression to moderate or severe malnutrition. Its scalability across healthcare tiers supports health system resilience, ensuring nutritional surveillance becomes integral to routine child health care.

## 7 Conclusion

The PMSA-2025 represents a meaningful step toward harmonizing pediatric nutritional screening across the Middle East. By combining clinical history, growth patterns, and behavioral cues into a concise, standardized framework targeting children aged 1–10 years with WHZ between −1 and −2 SD, it enables earlier identification of at-risk children in outpatient and community settings. The structured three-round Delphi methodology, I-CVI-based agreement quantification, and multidisciplinary panel composition provide a sound content validity foundation for the tool. Prospective validation across sensitivity, specificity, predictive values, and reliability metrics is planned. With its practicality, adaptability, and preventive orientation, the PMSA-2025 is hypothesized to strengthen early nutrition surveillance and promote timely intervention, pending empirical confirmation through the planned validation study.
